# Deepvirusclassifier: a deep learning tool for classifying SARS-CoV-2 based on viral subtypes within the coronaviridae family

**DOI:** 10.1186/s12859-024-05754-1

**Published:** 2024-07-05

**Authors:** Karolayne S. Azevedo, Luísa C. de Souza, Maria G. F. Coutinho, Raquel de M. Barbosa, Marcelo A. C. Fernandes

**Affiliations:** 1 InovAI Lab, nPITI/IMD, Federal University of Rio Grande do Norte, Natal, RN 59078-970 Brazil; 2 Bioinformatics Multidisciplinary Environment (BioME), Federal University of Rio Grande do Norte, Natal, RN 59078-970 Brazil; 3 Department of Pharmacy and Pharmaceutical Technology, University of Seville, 41012 Seville, Spain; 4 Department of Computer Engineering and Automation (DCA), Federal University of Rio Grande do Norte, Natal, RN 59078-970 Brazil

**Keywords:** SARS-CoV-2, Coronaviridae, Viral classification, Deep learning

## Abstract

**Purpose:**

In this study, we present DeepVirusClassifier, a tool capable of accurately classifying Severe Acute Respiratory Syndrome Coronavirus 2 (SARS-CoV-2) viral sequences among other subtypes of the coronaviridae family. This classification is achieved through a deep neural network model that relies on convolutional neural networks (CNNs). Since viruses within the same family share similar genetic and structural characteristics, the classification process becomes more challenging, necessitating more robust models. With the rapid evolution of viral genomes and the increasing need for timely classification, we aimed to provide a robust and efficient tool that could increase the accuracy of viral identification and classification processes. Contribute to advancing research in viral genomics and assist in surveilling emerging viral strains.

**Methods:**

Based on a one-dimensional deep CNN, the proposed tool is capable of training and testing on the Coronaviridae family, including SARS-CoV-2. Our model’s performance was assessed using various metrics, including F1-score and AUROC. Additionally, artificial mutation tests were conducted to evaluate the model’s generalization ability across sequence variations. We also used the BLAST algorithm and conducted comprehensive processing time analyses for comparison.

**Results:**

DeepVirusClassifier demonstrated exceptional performance across several evaluation metrics in the training and testing phases. Indicating its robust learning capacity. Notably, during testing on more than 10,000 viral sequences, the model exhibited a more than 99% sensitivity for sequences with fewer than 2000 mutations. The tool achieves superior accuracy and significantly reduced processing times compared to the Basic Local Alignment Search Tool algorithm. Furthermore, the results appear more reliable than the work discussed in the text, indicating that the tool has great potential to revolutionize viral genomic research.

**Conclusion:**

DeepVirusClassifier is a powerful tool for accurately classifying viral sequences, specifically focusing on SARS-CoV-2 and other subtypes within the Coronaviridae family. The superiority of our model becomes evident through rigorous evaluation and comparison with existing methods. Introducing artificial mutations into the sequences demonstrates the tool’s ability to identify variations and significantly contributes to viral classification and genomic research. As viral surveillance becomes increasingly critical, our model holds promise in aiding rapid and accurate identification of emerging viral strains.

## Introduction

One particular virus has made of attention of the entire world, the severe acute respiratory syndrome coronavirus 2 (SARS-CoV-2). The virus belongs to the family Coronaviridae, which contains one of the largest viral genomes, ranging from $26\text {,}000$ base pairs (bp) to $31\text {,}700$ bp and is well known for infecting animals and humans [1].Viruses from the same family have similar genetic and structural characteristics, which makes the classification process more challenging. This is especially true considering that the selection or extraction of resources is essential to carry out such differentiation. Furthermore, viruses can undergo recombination events where genetic material from different viruses combines, blurring the lines between viral families. The SARS-CoV-2 causes the COVID-19 disease, which has caused the death of thousands of people worldwide due to its high virulence rate in conjunction with your rapid spread [2, 3]. The novel and timely classification systems are necessary for more insights into the evolution of underlying mechanisms of increased epidemicity and enhanced virulence compared to related lineages [4, 5].

Classifying and identifying viruses remains a crucial and relevant task, even with the end of the pandemic. It is a widely applied task by many scientists worldwide. Virus classification is essential in several contexts, including areas related to genomics and viral surveillance. Furthermore, it supports the control, prevention, and treatment of future complications that these agents may cause in a population. This knowledge is valuable for the development of treatments, therapies, and vaccines for both known and emerging viruses [6]. This activity assigns a certain sequence to a specific group based on known genomic sequences which share common characteristics and traits [7]. The conventional methods for characteristics extraction of the virus are based on sequence alignment [8, 9]. Alignment-based techniques search for regions of similarity between biological sequences from a previously characterized reference sequence. These techniques can also be used for viral identification [7]. Alignment-based techniques are used in algorithms like Basic Local Alignment Search Tool (BLAST) [10], Megan alignment tool (MALT) [11], FASTQ preprocessor (FASTP) [12], ClustalW [13] and USEARCH [14]. However, these methods have some limitations: low accuracy and limited genomic sequence length used [8, 15]. The use of long genomic sequences implies a high computational cost due to the nature of the problem [16]. Works presented in [7, 8] draw attention to the evidence that alignment-based methods are not quite satisfactory when applied to genomes susceptible to large genetic variations, which is the case of the vast majority of the viruses. Furthermore, due to the high computational cost involved, alignment-based methods make it impossible to analyze a large number of complete genomes and in many cases, the structures need to be homologous [16]. In order to minimize these problems, free-alignment (FA) techniques emerged, which are based on features from linear algebra, information theory and statistical mechanics to calculate the similarity or distance between sequences [7, 8].

According to [7, 17, 18], to provide the best results, the viral classification based on free-alignment algorithms uses the artificial intelligence approach based on machine learning (ML) techniques to perform the feature extraction of the genomic sequences. Moreover, alignment-free techniques encompass methods that explore new forms of representation of input data by patterns identified in genomic data, as suggested by the works [15, 19, 20].Recent studies indicate that ML algorithms and techniques have been widely used in research related to genomics, including viral classification, for offering a set of methods capable of identifying highly complex patterns in an automated, efficient way and with the minimal human intervention [21, 22]. Works in the literature show that machine learning based on Deep Learning (DL) techniques provides excellent results for genomic sequences applications, including classification problems [23, 24].

Mottaqi [22] and Lalmuanawma [25] show that among many ML algorithms, the Convolutional Neural Networks (CNN) have been frequently used for data analysis based on genomic sequence for their ability to extract intrinsic characteristics of the sequences and present promising results in their applications. However, most of these tools and techniques use genomic sequences of limited length or are aimed at other purposes such as protein prediction [26, 27].

Fabijańska proposes a deep viral genome classifier, named VGDC (Viral Genome Deep Classifier), able to identify viral subtypes from different families such as dengue, hepatitis B and C, HIV-1, and influenza A presented F1-score between 0.85 and 1 [28]. Tampuu et al. presented an architecture to recognize the presence of viruses by the raw metagenomic contigs of various human samples. The methodology proposed was named ViraMiner and made use of two CNNs. They reached a Receiver Operating Characteristic (AUROC) curve of 0.923 [29].

The work presented by Whata et al. used a CNN and a Bi-LSTM (bi-directional long short-term memory), which he called CNN-Bi-LSTM (convolutional neural network bidirectional long short-term memory). This model achieved a classification accuracy of $99.95\%$, AUC of $100.00\%$, specificity of $99.97\%$, and sensitivity of $99.97\%$ as from 34 sequences from the SARS-CoV-2 virus and 295 samples from other viruses of the same family [30].

The study presented by Adetiba et al. used a CNN to perform a multiclass classification of genomic sequences of three viral subtypes, MERS-CoV (Middle East Respiratory Syndrome CoV), SARS-CoV (Severe Acute Respiratory Syndrome CoV), and SARS-CoV -2 (Severe Acute Respiratory Syndrome Coronavirus 2). The authors used the GSP (Genomic Signal Processing) technique to transform the genomic sequences into RGB images and later applied them to a CNN, using only 300 samples for training. The model obtained an accuracy of $95\%$ for MERS-CoV, $95\%$ for SARS-CoV, and $95\%$ for SARS-CoV-2, titled by the authors DeepCOVID-19 [31].

Classification between SARS-CoV-2, MERS-CoV, SARS-CoV, hepatitis-A, dengue, and influenza was proposed by Gunasekaran et al. Therefore, the authors use the CNN, CNN-LSTM, and CNN-Bidirectional LSTM architectures with *k*-mers to verify which architectures present better performance. According to the tests performed, it was observed that CNN and CNN-Bidirectional LSTM with *k*-mers offered the highest accuracy metrics, reaching $93.16\%$ and $93.13\%$, respectively [32]. A neural network called miRNA proposed by Lopez-Rincon et al. was applied at viral classification. The architecture has a few layers and was also used to classify viruses from the Coronaviridae family. This model showed an accuracy of $98\%$, specificity of 0.9939, and sensitivity of 1.00 [24].

Several viral genomic sequences of different sizes were analyzed by [33], which used the area under the receiver operating characteristic (AUROC) as their performance metric. The research obtained AUROC values of 0.95, 0.93, 0.97, and 0.98, for the genomic sizes 300, 500, 1000, and 3000 bp, respectively. The architecture used was called DeepVirFinder and consists of a CNN of multiple layers [33].

Given this context, the present work aims to present a technique capable of classifying the Coronaviridae family’s viruses and recognizing the SARS-Cov-2 virus. That approach uses the CNN that receives complete genomic sequences of cDNA as input, codified by the one-hot-encoding technique. The proposed method has high metrics and has been tested with over 10,000 complete SARS-CoV-2 sequences. Thus, this work makes the following specific contributions:

- Develop an alignment-free method to classify SARS-CoV-2 sequences between viruses from the same family, well known in the literature.
- Develop a deep learning algorithm that can efficiently classify the complete cDNA sequences of the virus.
- Comparison of the performance of the proposed model with the BLAST algorithm, recognized as the gold standard among alignment-free techniques, in terms of the number of samples found or correctly classified and the processing time taken by both tools to present their results.
- Utilization of a DL technique to analyze large datasets, enabling the efficient classification of numerous viral sequences in a short amount of time.
- Reduced computational cost when classifying many sequences compared to traditional established alignment-free methods.
- Use of partially mutated cDNA sequences to test the generalization and efficiency of the model in covering future mutations that may occur in the virus.

## Results

### Training and validation

**Fig. 1:**
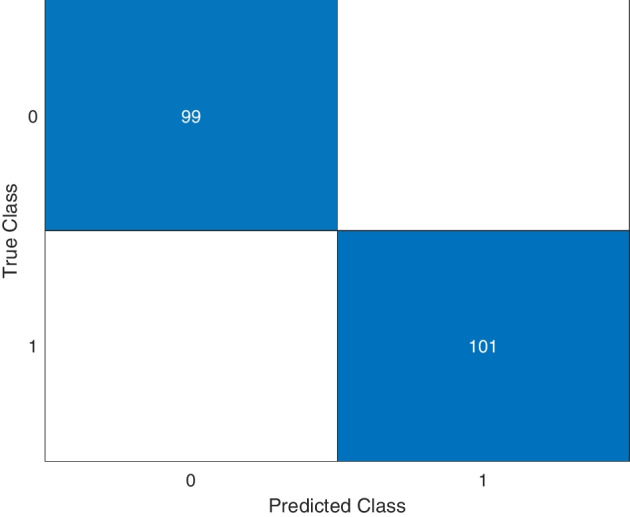
Confusion matrix of the proposed approach for the classification problem of distinguishing between SARS-CoV-2 and Non-SARS-CoV-2 samples. Non-SARS-CoV-2 samples are represented by label 0, and SARS-CoV-2 samples are represented by label 1. The model is capable of correctly classifying all samples according to their respective classes

**Fig. 2:**
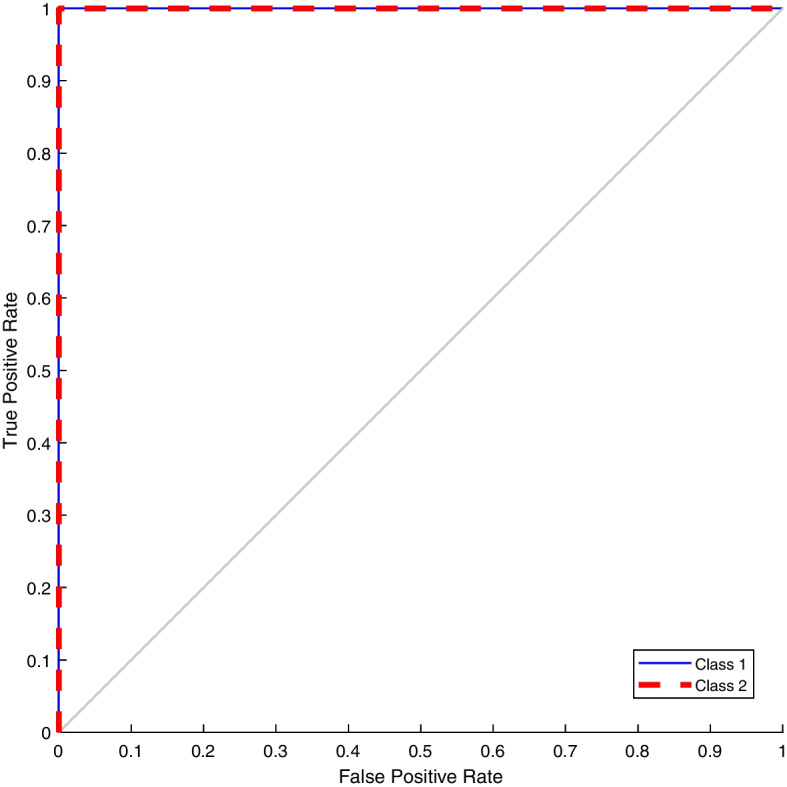
AUROC curve for classification of SARS-CoV-2 and Non SARS-CoV-2

As mentioned in “Database and data balancing” section, the dataset used for training the network comprises 501 samples referring to the Non-SARS group and receiving label 0 and 501 samples from SARS, in which they obtained label 1. In this way, we obtained a training set balanced and homogeneous consisting of 1002 samples. Cross-validation was used to train and validate the classification model (see “CNN architecture and parameters” section). The performance metrics for the *k*-fold ($k=5$) cross-validation corresponded to the average between all the values obtained in each fold. The classification results of validation (after training) were presented through the confusion matrix (see Fig. 1), the AUROC (see Fig. 2), and measured by the sensitivity, specificity, precision, accuracy, and F1-score metrics (see Table 1). As a result, the model results in maximum performance values for the training and validation sets, as shown in Table 1.

**Table 1:** Performance metrics results for the classification of SARS-Cov-2 from the architecture proposed in this work for the validation set

Metrics	Performance
Sensitivity	$100\%$
Specificity	$100\%$
Precision	$100\%$
Accuracy	$100\%$
F1-score	1

Figure 1 presents the results of the mean classification of the samples referring to the validation set (SARS-CoV-2 and Not SARS-CoV-2) and shows that for all subsets, all sequences were correctly grouped according to their respective class. The ROC curve for this problem is shown in Fig. 2 and presents sensitivity and specificity values equal to $100\%$, according to Table 1.

**Fig. 3:**
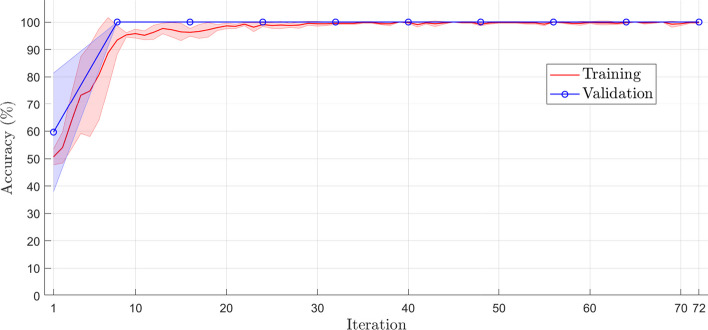
The learning curve of training and validation accuracy of the training set using fivefold cross-validation

**Fig. 4:**
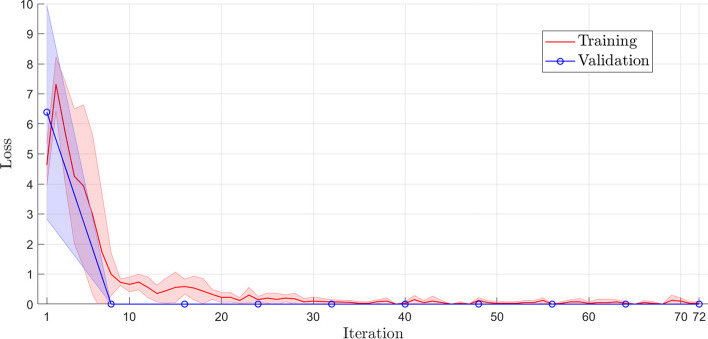
The learning curve of training and validation loss of the training set using fivefold cross-validation

Figures 3 and 4 illustrate the training and validation learning curve for accuracy and loss, respectively. Each iteration point represents the mean and standard deviations of the fivefold cross-validation. The accuracy learning curve of training and validation (see Fig. 3) corroborates with the results presented in Table 1, and these curves show that the model does not suffer from overfitting (high variance) or underfitting (high bias). Furthermore, the reduced difference (almost zero) between the training and validation curves consolidates the absence of overfitting. The training was concluded after 10 epochs with 72 iterations, as shown in Figs. 3 and 4. It is observed that the error was stabilized after the 30th iteration (see Fig. 4).

### SARS-Cov-2 prediction tests

Similar to the methodology used in [16], two tests were performed to evaluate the SARS-Cov-2 prediction of the proposed deep learning model after training. The tests were composed of samples not used in the training stage, that is, samples that remained from the initial dataset belonging to the SARS-CoV-2 virus (see “Pre-processing and data mapping” section). The tests, called Prediction test 1 and Prediction test 2, are described below.

#### Prediction test 1

Of the remaining $16\text {,}891$ SARS-CoV-2 samples from the initial dataset, $12\text {,}000$ were randomly chosen to compose this experiment. These samples obtained label 1 indicating that they were SARS-CoV-2. The objective of this experiment was to test the model for identifying SARS-CoV-2.

#### Prediction test 2

For this experiment, $10\text {,}000$ samples of SARS-CoV-2 were used (of the remaining $16\text {,}891$ SARS-CoV-2 samples from the initial dataset), in which they were divided into two groups, each with 5000 samples. In one of these groups, we applied the artificial mutation method discussed in “Artificial mutation technique” section to investigate the architecture’s sensitivity and robustness to possible mutations in the SARS-CoV-2 virus. In this way, a group was created with 5000 samples of the SARS-CoV-2 virus, which suffered artificial mutations, and another group, also with 5000 samples, which did not undergo any mutation. The artificial mutation strategy used $V_{max} = 31\text {,}029$ and $\gamma = 5\%$, i.e., $N_{\text {mut}}=1551$ nucleotides have changed per sequence.

#### Prediction test results

The results of Prediction tests 1 and 2 are shown in Table 2. For prediction test 1, $11\text {,}996$ were correctly classified to their respective group (SARS-CoV-2), and only 4 samples were not classified correctly, reaching $99.99\%$, $100\%$, $99,94\%$, and $99,96\%$ for the sensitivity, precision, F1-score, and accuracy, respectively. As described above, prediction test 2 verified the ability of the trained model to classify SARS-CoV-2 samples even after changing their genomic structure through the artificial mutation technique in half of the dataset samples. Even applying modifications to the sequences, the model is quite sensitive to possible mutations that the sequences may suffer, reaching a sensitivity value of $99.77\%$. This result strongly attests to the model’s ability to generalize, given that, even with the samples changing, the network can identify who is SARS-CoV-2 through low false negative results (accuracy about $99.96\%$).

**Table 2:** Results associated with prediction tests 1 and 2

Pt	Sensitivity (%)	Precision (%)	F1-score	Accuracy (%)
Pt-1	99.99	100	0.94	99.96
Pt-2	99.77	100	0.88	99.96

The results obtained through the experiments carried out and detailed in “Pre-processing and data mapping” section, are promising, consistent with the performance obtained in the network training phase. Furthermore, the sensitivity and precision values derived from the set of experiments remain high regardless of the class labels, which is very important, considering that high rates of false negatives directly corroborate the increase in infected people. The biological implications of these results are significant, as they showcase the robustness and high accuracy of the model in detecting SARS-CoV-2 even in the presence of artificial mutations. This underscores the model’s potential for practical applications in viral detection and classification, with implications for disease diagnosis and management. The high sensitivity of the tool is crucial in virus detection, as it minimizes the risks of false positives, ensuring reliable virus identification. High precision reduces unnecessary alerts or classification errors, which can have biological and public health consequences as viruses undergo mutations over time. A model that remains sensitive to these changes is invaluable for real-world applications, especially in the detection of new viral strains. The results obtained with this tool demonstrate the model’s resilience, high precision, and potential for practical applications in viral detection and classification, supporting diagnosis, disease management, and the detection of new viral variants. Finally, the proposed model’s characteristics and results will be compared and discussed with works found in the literature below.

## Methods

**Fig. 5:**
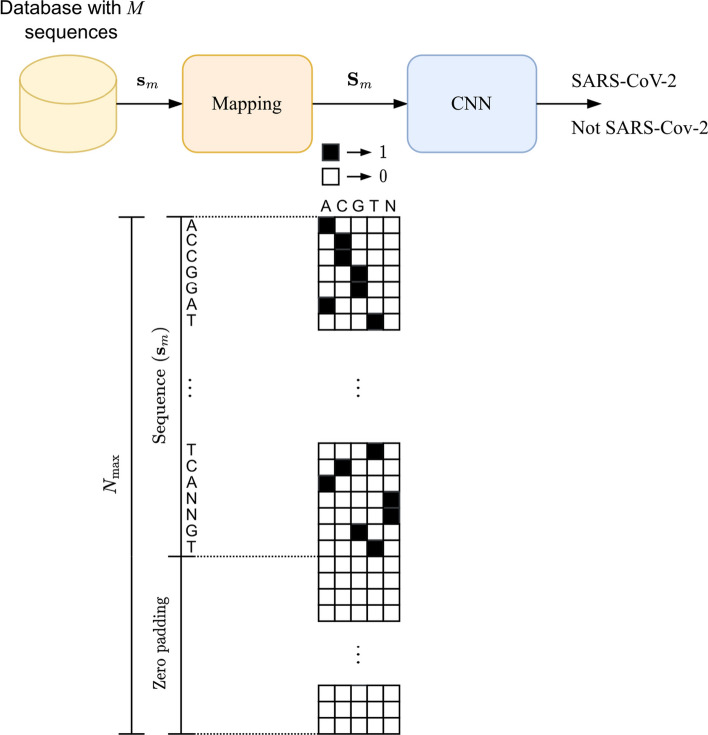
Overview of the proposed technique

The viral classification tool proposed in this work utilized genomic data from the cDNA of nine viral subtypes belonging to the Coronaviridae family, including SARS-CoV-2. The dataset underwent preprocessing, including balancing, transforming, and mapping viral sequences (see “Pre-processing and data mapping” section) to construct a homogeneous and balanced dataset. Subsequently, the CNN trained and processed the data, capable of extracting intrinsic features from the sequences, providing us with the classification result as either SARS-CoV-2 or non-SARS-CoV-2. Figure 5 below displays the flowchart of activities.

### Database and data balancing

The National Genomics Data Center (NGDC) provides open and free access to a set of database resources that have the resources of the New Coronavirus 2019 Data Resource - 2019nCoVR. The 2019nCoV maintains daily updates and brings together a comprehensive collection of genomic sequences and clinical information, not only about SARS-CoV-2 but also regarding other viruses that belong to the coronaviridae family worldwide and from other traditional repositories, such as the National Center for Biotechnology Information - NCBI [34]. The 2019nCoV was the chosen repository to download the dataset. Sequences belonging to the coronaviridae family were selected, whose size ranges from 25,000 to 35,000 bp, covering the size of all viruses in the family without losing any crucial genetic information. The selected host was the Homo Sapiens. The download of the dataset used in this research was carried out in August 2020, when the variants of concern were not yet available.

**Fig. 6:**
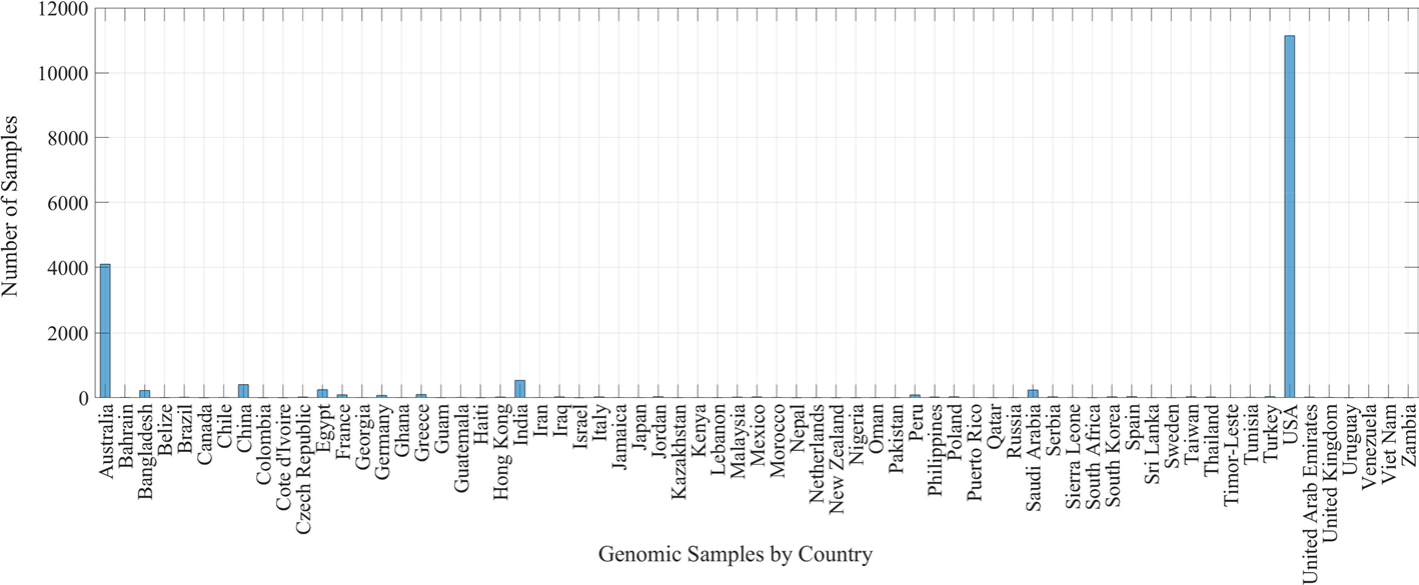
Countries that contain genomic samples of the coronaviridae family in the database

The database used is formed by $17\text {,}893$ genomic sequences of nine types of viruses of the coronaviridae family, coming from 62 different countries. Figure 6 shows all countries with genomic samples on the database. It is observed that the United States has the highest number of sequences, followed by Australia, India, and China. From the $17\text {,}893$ samples, $17\text {,}392$ belong to the SARS-CoV-2 virus $97.2\%$ of all), of which $11\text {,}140$ are coming from the United States ($62.25\%$ of all).

The data used for viral classification are cDNA sequences, whose length varies from 26,342 to 31,029 bp. Table 3 summarizes some properties related to viral subtypes present in the database. The BetaCoronaVirus shows the most extensive sequence length among all virus subtypes, varying between 31,029 and 30,536 bp. In addition to having the same sequence length (30,499 bp), the *CoronaVirus cya-BetaCov/2019*, *CoronaVirus cyb-BetaCov/2019*, and *CoronaVirus cyc-BetaCov/2019* are the viruses that have the smallest amount of samples in the database. They are long genomic samples and very similar viruses, so a robust model is required to provide the appropriate classification [28].

**Table 3:** Viral subtypes on the database created for this work

Virus	Number of samples	Minimum sequence length	Maximum sequence length
BetaCoronaVirus	140	$30\text {,}536$	$31\text {,}029$
CoronaVirus cya-BetaCov/2019	1	$30\text {,}499$	$30\text {,}499$
CoronaVirus cyb-BetaCov/2019	1	$30\text {,}499$	$30\text {,}499$
CoronaVirus cyc-BetaCov/2019	1	$30\text {,}499$	$30\text {,}499$
HCoV-229E	27	$26\text {,}592$	$27\text {,}307$
HCoV-HKU11	18	$29\text {,}367$	$29\text {,}983$
HCoV-NL63	55	$27\text {,}302$	$27\text {,}832$
MERS-CoV	258	$29\text {,}267$	$30\text {,}150$
SARS-CoV-2	$17\text {,}392$	$26\text {,}342$	$28\text {,}784$

As shown in Table 3, the largest amount of samples in the database belong to the SARS-CoV-2 virus, which causes the COVID-19 disease, followed by the MERS-CoV virus. In this context, it was necessary to balance the data to improve the network’s performance and avoid problems such as Overfitting due to the disproportion of samples from the other viruses.

**Fig. 7:**
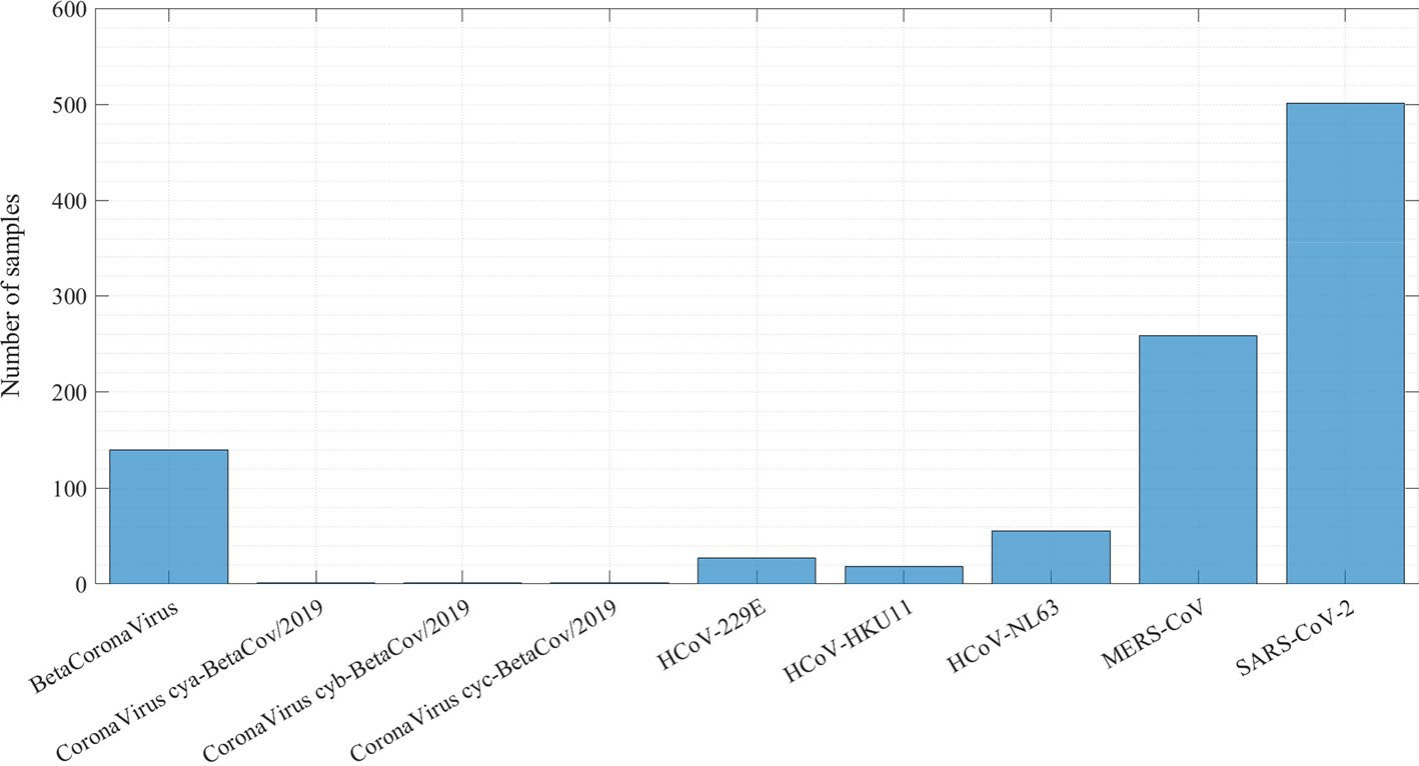
Dataset of all viral subtypes after the data balancing process

**Fig. 8:**
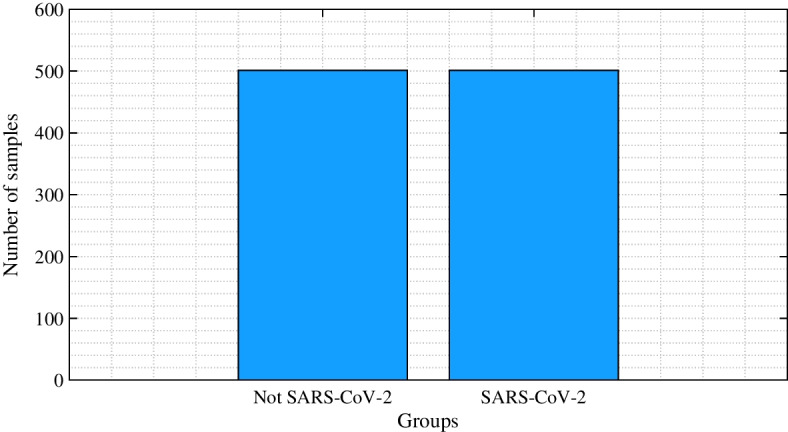
Dataset after balancing the samples according to their groups

The dataset was divided into two groups: non SARS-CoV-2 and SARS-CoV-2, as illustrated in Fig. 7. The non SARS-CoV-2 group comprises eight viral subtypes different from the SARS-CoV-2 virus, totaling 501 samples. Therefore, 501 samples were taken from all countries that presented genomic sequences of the SARS-CoV-2 virus randomly and uniformly, guaranteeing diversity and representativeness of each viral subtype in the training and validation sets, as illustrated in Fig. 8. The dataset used for the training and validation phases contains 1002 samples in total. The samples were labeled by 0 and 1, where 0 is associated with the non SARS-CoV-2 samples, and 1 is related to the SARS-CoV-2 samples. Part of the remaining genomic samples was used to test the performance of the network.

### CNN architecture and parameters

Based on the length of the sequences in the database presented in Table 3, it appears that the most prolonged sequences correspond to BetaCoronaVirus. Therefore, all genomic sequences will have the same length ($N_{\text {max}} = 31\text {,}029$) to be processed by CNN. Then, for each *m*th sample, the CNN receives as entry 5 channels of dimension $31\text {,}029 \times 1$. As described in “Pre-processing and data mapping” section, this strategy allows all *M* viral sequences have the same length.

**Fig. 9:**
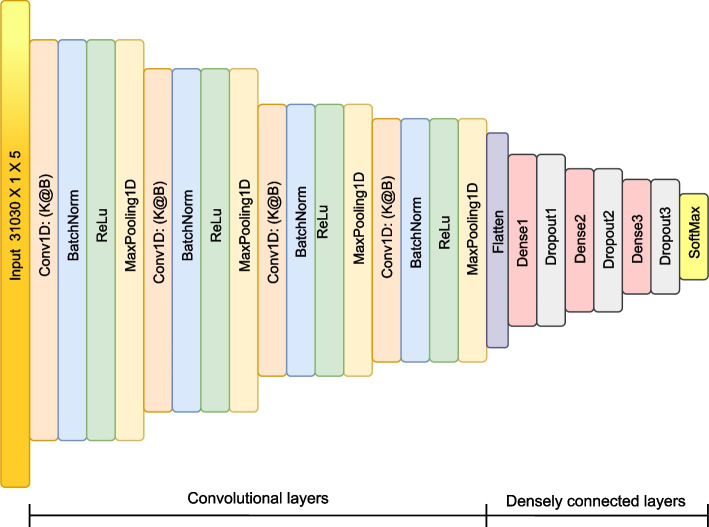
CNN used for the viral classifier proposal presented in this work

The CNN used in this work comprises twenty-six layers, divided into 1D (one-dimensional) convolutional layers and fully connected layers. The 1D convolutional layers are responsible for extracting characteristics of the cDNA genomic sequences, and the fully connected layers are responsible for classifying the data extracted from the previous layers, generating a total of $14\text {,}545\text {,}426$ parameters across all layers, as shown in Table 4. Figure 9 details the CNN architecture used in the appropriate viral classifier for the database described in “Database and data balancing” section.

**Table 4:** CNN architecture used in this work with four convolutional layers and four fully connected layers

Layers	Description	Values
1	Input ($L \times 1 \times 5$)	$N = 31\text {,}030$
2	Conv1d ($K_{1}$@$B_{1}$)	$K_{1} = 256$ and $B_{1} = 8$
3	BatchNorm	–
4	ReLU	–
5	MaxPool1D ($P_{s}$)	$P_{s}$ = 8
6	Conv1D ($K_{2}$@$B_{2}$)	$K_{2} = 64$ and $B_{2} = 16$
7	BatchNorm	–
8	ReLU	–
9	MaxPool1D ($P_{s}$)	$P_{s} = 16$
10	Conv1D ($K_{3}$@$B_{3}$)	$K_{3} = 32$ and $B_{3} = 8$
11	BatchNorm	
12	ReLU	–
13	MaxPool1D ($P_{s}$)	$P_{s}$ = 32
14	Conv1D ($K_{4}$@$B_{4}$)	$K_{4} = 32$ and $B_{4} = 64$
15	BatchNorm	–
16	ReLU	–
17	MaxPool1D ($P_{s}$)	$P_{s} = 64$
18	Flatten	–
19	Dense1 ($P_{1}$)	$P_{1} = 64$
20	Dropout ($a_{1}$)	$a_{1} = 0.4$
21	Dense2 ($P_{2}$)	$P_{2} = 32$
22	Dropout ($a_{2}$)	$a_{2} = 0.4$
23	Dense3 ($P_{3}$)	$P_{3} = 16$
24	Dropout ($a_{3}$)	$a_{3} = 0.4$
25	Dense4 ($P_{4}$)	$P_{4} = 2$
26	Softmax	2 Classes

The CNN comprises four convolutional layers, followed by a normalization layer and the activation function ReLu (Rectified Linear Unit). The MaxPool function is applied after each activation layer, with windows ranging in size from 8, 16, 32 and 64. In addition to the convolutional layers, the CNN structure contains four fully connected layers with 64, 32, 16, and 2 neurons, respectively. The number of neurons in the last layer corresponds to the number of classes to be classified, followed by the softmax function that will output the probability that each sequence belongs to a specific class.

The cross-validation *k*-fold was used to evaluate the proposed model, where *k* refers to the number of subsets, or folds, into which the dataset will be divided. We defined the value of $k=5$ so that the dataset will be divided into five subsets, each fold containing 201 samples. In the cross-validation scheme, $k-1$-folds are used for model training (801 samples), and onefold is used for model validation (201 samples), totaling 1002 samples.The optimizer chosen for updating the network weights was the adam (Adaptive Moment Estimation), whose learning rate was 0.001 (see Table 5). An optimizer is a function that aims to reduce the error between the results obtained by a model concerning the desired results. Among the various optimizers, adam is one of the most used in the literature, especially in deep learning. This optimizer is indicated in problems that involve a large amount of data or parameters because it is easy to implement, has a low computational cost, and requires a low amount of memory [35]. The training converged in approximately 10 epochs. Given the nature of the problem and through tests and works found in the literature, a mini-batch of size 128 was applied due to the number of samples and training parameters as recommended in [28]. The parameters used in the architecture training phase are shown in Table 5. A mini-batch of 128 was used based on the long length of the viral genomes and the large number of samples used to train the model. Other parameters were adjusted to decrease the training time and the loss function as recommended in [18, 24, 28]. The training converged in approximately 10 epochs with 72 iterations (see Figs. 3, 4 in “Training and validation” section).

The proposed CNN model was based on prior work found in the literature [24, 28]. However, modifications were made to the network to achieve the best model performance given the type and quantity of data used. The proposed architecture brings some potential innovations, such as the method for standardizing the length of viral genomic sequences, enabling effective handling of sequences of varying lengths. This can be particularly important when dealing with real-world data, where sequences may have different lengths, which can influence the choice of parameters and network size to achieve maximum performance metrics. While most CNN architectures operate in two or three dimensions, this work utilized a one-dimensional CNN, which has reduced computational complexity compared to the 2D or 3D CNNs widely used in the literature.

The proposed CNN model was based on prior work found in the literature [24, 28]. However, modifications were made to the network to achieve the best model performance, given the type and quantity of data used. The proposed architecture brings some potential innovations, such as the method for standardizing the length of viral genomic sequences enabling effective handling of sequences of varying lengths. This can be particularly important when dealing with real-world data, where sequences may have different lengths, influencing the choice of parameters and network size to achieve maximum performance metrics. While most CNN architectures operate in two or three dimensions, this work utilized a one-dimensional CNN, which has reduced computational complexity compared to the 2D or 3D CNNs widely used in the literature.

**Table 5:** Hyperparameters used in the training phase of the proposed architecture

Hyperparameters	Values
Mini-batches	128
MaxEpochs	12
InitialLearnRate	0.001
Optimizer	Adam

### Pre-processing and data mapping

The methodology used in this work can be divided into two stages: (1) pre-processing and data mapping; (2) methods to verify and test the model’s generalization. For CNN to perform feature extraction and classification, it is necessary to pre-process the data, which involves converting the nucleotides of the genomic sequences, represented by the characters (A, C, G, T, N), into numerical data, precisely ones and zeros. Once encoded, the data will be mapped into vectors of a dimension and depth of 5, using the one-hot-encode technique to be presented to CNN, indicating whether or not it is SARS-CoV-2.

The Fig. 5 illustrates the overview of the technique proposed in this work. Considering a database with *M* samples of DNAc viral sequences, each *m*th sample, $\textbf{s}_m$ is mapped in a characteristic matrix, $\textbf{S}_m$, that will be processed by the CNN. The CNN provides a binary classification in which the SARS-CoV-2 will be identified or not.

Each *m*th sample of viral sequence de entrada is expressed by

1 $$\begin{aligned} \textbf{s}_m = [s_{1,m},\dots ,s_{N_m,m}] \end{aligned}$$

where each *i*th element of a *m*th sample, $s_{i,m}$ represents a possible nucleotide of a set $S \in \{\text {A},\text {C},\text{ G },\text {T}\}$, and $N_m$ is the length of the *m*th viral sequence sample. Each element of *S* corresponds to one of the nitrogenous bases Adenine (A), Cytosine (C), Guanine (G) and Thymine (T).

The characteristic matrix associated with the *m*th sample, $\textbf{s}_m$, is constructed by the one-hot encode technique, which can be expressed as

2 $$\begin{aligned} \textbf{S}_m = \left[ \begin{array}{ccc} a_{1,1,m} &{}\quad \dots &{}\quad a_{1,5,m} \\ \vdots &{}\quad \ddots &{}\quad \vdots \\ a_{N_{\text {max}},1,m} &{}\quad \dots &{}\quad a_{N_{\text {max}},5,m} \end{array} \right] \end{aligned}$$

where

3 $$\begin{aligned} a_{i,j,m} = {\left\{ \begin{array}{ll} 1 &{}\text{ for } \quad j=1 \,\, \& \,\, s_{i,m} = \text {A} \\ 1 &{}\text{ for } \quad j=2 \,\, \& \,\, s_{i,m} = \text {C} \\ 1 &{}\text{ for } \quad j=3 \,\, \& \,\, s_{i,m} = \text {G} \\ 1 &{}\text{ for } \quad j=4 \,\, \& \,\, s_{i,m} = \text {T} \\ 0 &{} \text{ for } \quad \forall j \,\, \& \,\, s_{i,m} \notin S \end{array}\right. } \end{aligned}$$

and $N_{\text {max}}$ is the size of the largest sequence among all the *M* viral sequence samples, that is, $N_{\text {max}} = \max \left\{ N_1,\dots ,N_M \right\}$. So, the characteristic matrix has the same dimension ($N_{\text {max}} \times 5$) for all the *M* samples of viral sequences. If the size of the *m*th sequence is less than the maximum sequence ($N_m < N_{\text {max}}$), $N_{\text {max}} - N_m$ zeros are inserted (zero padding).

Before entering into the CNN, the characteristic matrix of each *m*th sample, $\textbf{S}_m$, is transformed into a matrix of dimension $N_{\text {max}} \times 1 \times 5$, expressed as

4 $$\begin{aligned} \textbf{B}_m = \left[ \begin{array}{ccc} \textbf{b}_{1,m}&\dots&\textbf{b}_{5,m} \end{array} \right] \end{aligned}$$

where

5 $$\begin{aligned} \textbf{b}_{j,m} = \left[ \begin{array}{ccc} b_{1,1,j,m} \\ \vdots \\ b_{N_{\text {max}},1,j,m} \\ \end{array} \right] \end{aligned}$$

which $b_{i,1,j,m} = a_{i,j,m}$. This transformation allows the CNN to process each *m*th sequence as an input formed by 5 channels of dimension vectors $\left( N_{\text {max}} \times 1 \right)$, $\textbf{ b}_{j,m}$.

### Artificial mutation technique

The artificial mutation process is initiated by searching for the maximum sequence length among the samples. So, for the set *H* of samples, $V_{max} = \max \left\{ N_1,\dots ,N_H \right\}$, where $N_i$ is the length of the sequences and $V_{max}$ is the length of the most extensive sequence. After this step, the insertion of zeros is performed in each *i*th sequence, $s_i$, where $N_i < V_{max}$. Each *i*th sequence is completed with zeros until filling the value of $V_{max}$, i.e., the amount of zeros entered for the *i*th sequence is $V_{max} - N_i$. After that, all the chosen *H* samples will have the same size, $V_{max}$. The artificial position mutation rate, $\gamma$, is defined at the end of this step. The value of $\gamma$ establishes the percentage of the number of nucleotides positions that will change, $N_{\text {mut}}$, which can be expressed as

6 $$\begin{aligned} N_{\text {mut}} = \left\lfloor \frac{\gamma \times V_{max}}{100} \right\rfloor . \end{aligned}$$

After the definition of the $N_{\text {mut}}$, the position of the $N_{\text {mut}}$ nucleotides that will be changed is randomly defined, which is stored in the vector $\textbf{k} _{\text {mut}} = \left[ k_1,\dots ,k_{N_{\text {mut}}} \right]$. From the position vector, $\textbf{k} _ {\text{ mut }}$, two methods are applied to change the selected nucleotides for artificial mutation. The first method was applied to the first half of the selected nucleotides, i.e., the positions $\left[ k_1,\dots ,k_{N_{\text {mut}}/2} \right]$, and the second method was used for the second half of the position vector $\left[ k_{N_{\text {mut}}/2+1}, \dots ,k_{N_{\text {mut}}} \right]$.

The first method changes the position of the nucleotides, considering the pairs, i.e.

7 $$\begin{aligned} \begin{aligned} \left[ k_1,k_2,\dots ,k_{N_{\text {mut}}/2-1},k_{N_{\text {mut}}/2} \right] \Rightarrow \\ \left[ k_2,k_1,\dots ,k_{N_{\text {mut}}/2},k_{N_{\text {mut}}/2-1} \right] \end{aligned}. \end{aligned}$$

Furthermore, the second method changes the nucleotide values of each *m*th sequence according to the $s_{k_i,m}$ position can be expressed by

8 $$\begin{aligned} s_{k_i,m} = {\left\{ \begin{array}{ll} \text {A} &{}\text{ if } \quad s_{k_i} = \text {T} \\ \text {T} &{}\text{ if } \quad s_{k_i} = \text {A} \\ \text {C} &{}\text{ if } \quad s_{k_i} = \text {G} \\ \text {G} &{}\text{ if } \quad s_{k_i} = \text {C} \\ \text {N} &{}\text{ if } \quad s_{k_i} = \text {T} \end{array}\right. }. \end{aligned}$$

It is important to note that the designations $s_{i,m}$ and $s_{k_i,m}$ refer to the same element, where $k_i$ identifies the exact position of the nucleotide that will undergo alteration in the sequence $s_{i,m}$.

## Discussion

### Blast comparison

The strategy proposed in this work was compared with the BLAST algorithm. The comparison obtained results associated with the correctness rate in the classification of sequences through various values of artificial position mutation rate (see “Artificial mutation technique” section) and the average processing time to classify these sequences. In the comparison, 34 sequences belonging to the Coronaviridae family were used (17 SARS-CoV-2 and 17 Not SARS-CoV-2) that did not participate in the deep learning training.

The BLAST software version 2.13.0 made available by the NCBI [34] was downloaded and installed locally. The BLAST software used a database of $6\text {,}180\text {,}834$ Betacoronavirus sequences (updated Sep 8, 2022) found in [34]. The database was also downloaded for local use. Using the BLAST software locally, accessing a local database allows a fairer comparison in terms of processing time with the deep learning strategy proposed in this work. The same computer used to run BLAST with its database was also used to train and run the CNN strategy. The computer has the following configurations: Intel(R) core(TM) i7-10700 CPU 2.9 GHz, 128 GBytes of RAM, 512 GBytes NVMe HD and an NVIDIA GeForce RTX 3060 GPU with 12 GBytes of RAM.

**Fig. 10:**
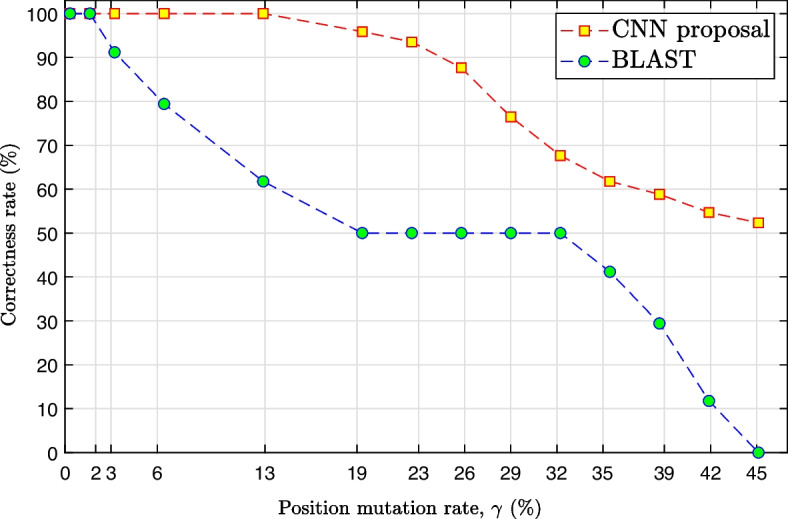
Comparison of the correctness rate between BLAST and CNN (proposed in this work) for a test set of 34 sequences according to the increase of the artificial position mutation rate, $\gamma$

Figure 10 presents the relationship between the artificial position mutation rate (see “Artificial mutation technique” section) applied in the 34 test sequences and the correctness rate (in percentage terms) of both the BLAST and the proposed CNN. It is possible to observe that up to $\gamma \approx 2\%$ ($N_{\text {mut}}\approx 620$ nucleotides), the correctness rate for BLAST and CNN-based strategy is the same, that is, $100\%$. However, for values of $\gamma > 2\%$, the correctness rate of BLAST drops rapidly to $50\%$, in which $\gamma \approx 19\%$ ($N_{\text {mut}}\approx 5895$ nucleotides). On the other hand, the proposal based on CNN has a correctness rate of $100\%$ up to $\gamma \approx 13\%$ ($N_{\text {mut}}\approx 4033$ nucleotides) and decays more slowly than BLAST, with $\gamma >13\%$. For $\gamma \approx 19\%$, a proposal based on CNN has a correctness rate of around $95.88\%$ and BLAST around $50\%$. For values of $\gamma$ between $\approx 32\%$ ($N_{\text {mut}}\approx 9\text {,}929$ nucleotides) and $\approx 45\%$ ($N_{\text {mut}}\approx 13\text {,}963$ nucleotides), the correctness rate of BLAST rapidly decays to zero while the proposal with CNN decays more slowly to $50\%$. Table 6 presents the values of correctness rate, artificial position mutation rate, $\gamma$, and the number of nucleotides that mutated, $N_{\text {mut}}$, for each point in the graphs shown in Fig. 10.

**Table 6:** Values of correctness rate, artificial position mutation rate, $\gamma$, and the number of nucleotides that mutated, $N_{\text {mut}}$, for each point in the graphs shown in Fig. 10

$\gamma$ ($\%$)	$N_{\text {mut}}$	BLAST	CNN
		Correctness rate ($\%$)	Correctness rate ($\%$)
0.32	100	100.00	100.00
1.61	500	100.00	100.00
3.22	1000	91.18	100.00
6.45	2000	79.41	100.00
12.89	4000	61.76	100.00
19.34	6000	50.00	95.88
22.56	7000	50.00	93.53
25.78	8000	50.00	87.65
29.01	9000	50.00	76.47
32.23	10,000	50.00	67.65
35.45	11,000	41.18	61.76
38.67	12,000	29.41	58.82
41.90	13,000	11.76	54.71
45.12	14,000	0.00	52.35

Table 7 presents the average processing time obtained for BLAST and CNN at each point presented in the graphs in Fig. 10. The data presented for CNN are the time required to perform the inference of the 34 test sequences, given that the training is performed only once. However, the time for training the CNN was approximately 341 s (around 6 min). It is possible to observe that CNN has a constant processing time while BLAST has a variable processing time that depends on the value of $\gamma$.

**Table 7:** Time processing, artificial position mutation rate, $\gamma$, and the number of nucleotides that mutated, $N_{\text {mut}}$, for each point in the graphs shown in Fig. 10

$\gamma$ ($\%$)	$N_{\text {mut}}$	BLAST	CNN
		Time processing (s)	Time processing (s)
0.32	100	$94\text {,}261.48$ ( $\approx 26.2$ h)	0.33
1.61	500	$94\text {,}261.48$ ( $\approx 26.2$ h)	0.35
3.22	1000	$93\text {,}202.74$ ( $\approx 25.9$ h)	0.39
6.45	2000	$92\text {,}172.83$ ( $\approx 25.6$ h)	0.45
12.89	4000	$91\text {,}176.66$ ( $\approx 25.3$ h)	0.68
19.34	6000	$64\text {,}122.58$ ( $\approx 17.8$ h)	0.68
22.56	7000	$24\text {,}587.36$ ( $\approx 6.8$ h)	0.68
25.78	8000	$68\text {,}17.63$ ( $\approx 1.9$ h)	0.68
29.01	9000	$44\text {,}35.43$ ( $\approx 1.2$ h)	0.68
32.23	$10\text {,}000$	2155.14 ( $\approx 0.6$ h)	0.68
35.45	$11\text {,}000$	1940.66 ( $\approx 0.5$ h)	0.68
38.67	$12\text {,}000$	1831.33 ( $\approx 0.5$ h)	0.69
41.90	$13\text {,}000$	1832.6 ( $\approx 0.5$ h)	0.69
45.12	$14\text {,}000$	1801.26 ( $\approx 0.5$ h)	0.68

For sequences with many mutations, $\gamma >25.78$ ($N_{\text {mut}}>8000$), BLAST has a faster response (shorter processing time) than for sequences with few mutations $\gamma <3.22$ ($N_{\text {mut}}<1000$). Sequences with many mutations allow BLAST to reduce the search space due to the high dissimilarity between the query sequence and the sequences stored in the base. On the other hand, when the value of g decreases, the BLAST processing time increases to obtain a better similarity value between the query sequence and the sequences stored in the base.

The gain in CNN processing time over BLAST is significant, being around 2600 times faster for $\gamma =45.12\%$ ($N_{\text {mut}}=14\text {,}000$) and $130\text {,}000$ times faster for $\gamma =0.32\%$ ($N_{\text {mut}}=100$). It is essential to point out that BLAST needs a database of sequences already stored to find or classify the viral genome, and with this, it needs to carry out a search procedure which can take a long time. CNN stores the information needed to classify the viral genome in its models after the training process. After training, the CNN performs only a simple inference process, not needing to perform a search and a database.

The proposed CNN model can be an excellent alternative and ally in the rapid virus classification process, given its high sensitivity in detecting changes in the virus structure (represented by random mutations in its nucleotides), corroborating SARS-Cov-2 surveillance. In addition, this model enables the analysis of more significant amounts of complete genomic samples, at a lower computational cost, compared to techniques that use alignment and even BLAST.

### State of the art comparison

The Tables 8 and 9 summarize a set of approaches from the main works found in the literature, and addressed in this article, that perform viral classification using CNNs and viral sequences as input data with the aim of maintain a fairer comparison with the proposed technique. Characteristics such as the number of layers and size of genomic sequences will be presented in Table 8.

**Table 8:** Comparison from the proposed architecture with related works

References	Codification	Layers	Sequence length
Fabijańska e Grabowski [28]	ASCII	30	3257–24,751 bp
Ren et al. [33]	One-hot encoded	6	150–3000 bp
Tampuu et al. [29]	One-hot encoded	2 CNNs with 7 layers each	300 bp
Lopez-Rincon et al. [24]	Assigned values from 0 to 1 to the channels	10	$31\text {,}029$
Proposed architecture	One-hot encoded	26	$31\text {,}029$

When applying longer sequences, the works presented in [28, 29, 33] had a considerable reduction in the performance of their models. This point implied the use of more extensive networks as in [28] and the reduction of sequence sizes as in works [29, 33].

Regarding [24], despite making use of complete genomic sequences and presenting a smaller number of layers, the author makes use of a small dataset for the training and validation of his model, which may lead to generalization problems and consequently on the performance of your network by presenting new samples. Table 9 compares the performance results of the proposed architecture with the available results of the models in Table 8.

Although it presents an architecture with many layers, the variation in the performance values of the VGDC architecture was observed as the size of the genomic sequences used in the network increased. Although it uses two convolutional branches, the ViraMiner tool achieved $92.3\%$ and $32\%$ of the sensitivity and precision values, even using relatively short sequences.

**Table 9:** Performance metrics comparison from the proposed architecture with related works

Ref.	Accuracy	Precision	Sensibility	Specificity	F1-score	AUROC
[28]	0.99–1	0.83–1	0.84–1	0.99–1	0.83–1	–
[33]	–	–	–	–	–	0.8635
						0.9210
						0.9496
						0.9668
[29]	0.90	0.90	0.32	–	–	0.923
[24]	0.985	0.98	1	0.9939	0.9797	0.92
This work	1	1	1	1	1	1

The DeepVirFinder architecture provided only the AUROC values obtained in its model, reaching the maximum value of $96.68\%$ for samples with 3000 bp. Despite having obtained the sensitivity value of $100\%$ and accuracy of $98\%$. The work presented by [24] obtained the AUROC value of $92\%$. The results obtained in the proposed model are superior for all architectures and performance metrics presented in Table 9, indicating the high performance and robustness of the model. The DeepVirusClassifier showcases a robust learning capacity, as demonstrated by its ability to achieve exceptional performance when tested on a large dataset comprising more than $10\text {,}000$ viral sequences. It maintains a sensitivity of over $99\%$ for sequences with fewer than 2000 mutations.

## Conclusion

Classification and prediction of viral sequences using deep neural networks (DNN) have shown great promise in recent years. This work proposes a tool, called DeepVirusClassifier, which uses a DNN-type CNN capable of classifying SARS CoV 2 through a binary classification based on complete genomic cDNA sequences among eight viral subtypes belonging to the same family. For this experiment, the cross-validation technique with k=5 folder was used, which reached maximum values in all evaluation metrics for the 960 samples used in training. More than 10,000 sequences were used to test the performance of the DNN after training. An artificial mutation technique was also used to test the generalizability of the model with sensitivity greater than 99% for less than 2000 mutations in the sequence. A test set consisting of 34 samples from the two classes experienced different position mutation rates and was processed by the model proposed in this work in conjunction with the BLAST algorithm to verify its performance in terms of accuracy rate according to the two classes. Taking into account results of accuracy and processing time, the proposed tool appears to be superior. To establish the superiority and practical applicability of our model, we carried out a comparative analysis with existing viral classification works in the literature, our results surpassed them. The proposed model was superior, indicating that the tool proposed in this work can be applied to classify viruses from the Coronaviridae family and viruses from different species. While the text primarily concentrates on classifying sequences from SARS-CoV-2 and the Coronaviridae family, the model architecture is versatile and has the potential to be adapted for classifying sequences from other viral families or applied to various sequence classification tasks. Our research signifies a substantial advancement in the field of viral sequence classification, opening the door to more precise and efficient tools in virology and bioinformatics and establishing itself as a reference for future research. DeepVirusClassifier significantly contributes as a foundation for early disease detection and diagnosis, genomic surveillance, and drug development, and even aids in identifying specific viral strains.

## Data Availability

The datasets generated and/or analysed during the current study are available in the Mendeley Data repository, https://data.mendeley.com/datasets/zmhsn2gz7w/1.
